# Multiple schwannomas of the facial nerve mimicking cervical lymphoma: a case report

**DOI:** 10.1186/s13256-021-03006-x

**Published:** 2021-08-20

**Authors:** Jan Philipp Kühn, Mathias Wagner, Alessandro Bozzato, Maximilian Linxweiler

**Affiliations:** 1 Department of Otorhinolaryngology, Head and Neck Surgery, Saarland University Medical Center, Kirrbergerstr. 100, building 6, 66421 Homburg, Germany; 2 Department of General and Surgical Pathology, Saarland University Medical Center, 66421 Homburg, Germany

**Keywords:** Multiple schwannomas, Facial nerve, Cervical lymphoma, Parotid gland

## Abstract

**Background:**

In this report, we describe the first case in literature of a patient with multiple schwannomas of the marginal mandibular branch of the facial nerve.

**Case presentation:**

A Caucasian patient presented with a sudden onset of left lower facial nerve palsy House–Brackmann score III for 1 month. Computed tomography imaging was performed to exclude a cerebral event and revealed multiple tumors within the left parotid gland. Duplex ultrasound and magnetic resonance imaging scans delineated multiple, hypoechoic tumors, round in shape and well defined without a hilar structure along the left mandible. For histological verification, a left-side partial parotidectomy and extirpation of an intraparotideal node was performed with use of a nerve-integrity monitor. Histomorphological analysis of the resected tissue revealed a benign schwannoma. Facial nerve function remained unchanged since the operation. The size of the nonresected tumors is currently monitored regularly by ultrasonography. Fibromatosis has been excluded.

**Conclusions:**

If multiple tumors occur in the parotid gland and the angle of the jaw, schwannomas need to be considered as a differential diagnosis. To plan the right diagnostic surgical intervention and prevent nerve damage, a thorough ultrasound examination is essential in preoperative diagnostic work-up for any suspicious lesion of the parotid gland and jaw region.

## Background

Schwannomas are benign nerve sheath tumors arising from Schwann cells of myelinated peripheral or cranial nerves. They are commonly located in the head and neck region and account for 25–45% of extracranial schwannomas [1, 2]. Schwannomas of the facial nerve are extremely rare, with less than 50 cases reported worldwide. They can affect any segment of the nerve, most commonly the geniculate ganglion. Most of the facial nerve Schwannomas are localized in the intratemporal region, and only 9% of cases involve a portion of the extratemporal segment [1, 2]. If they grow multifocally, an association with neurofibromatosis type I and II has to be considered [2]. To date, multiple schwannomas of peripheral nerves have been described affecting, for example, the nerves of the extremities [3–5].

In this report, we describe the first case in literature of a patient with multiple schwannomas of the marginal mandibular branch of the facial nerve.

## Case presentation

A 73-year-old female Caucasian patient presented at the Department of Otorhinolaryngology, Head and Neck Surgery of the Saarland University Medical Center (Homburg/Saar, Germany) with a sudden onset of left-sided nerve palsy of the lower facial nerve branches (House–Brackmann score III) characterized by slightly weak motion of the mouth with maximum effort, for 1 month. She denied weight loss, fevers, or night sweat. Her past medical history consisted of hypertension treated with metoprolol (47.5 mg 1–0–1), ramipril (2.5 mg 1–0–1), and hydrochlorothiazide (12.5 mg 1–0–0) as well as an ischemia of the right thalamus 2 years ago without any persisting neurological deficits. Additional medication on admission included aspirin for inhibition of platelet aggregation (100 mg 1–0–0). She used to work as a shop assistant and retired 5 years ago. She lives together with her husband in a small city and has two grown-up healthy children. She never smoked or drank alcohol.

**Fig. 1:**
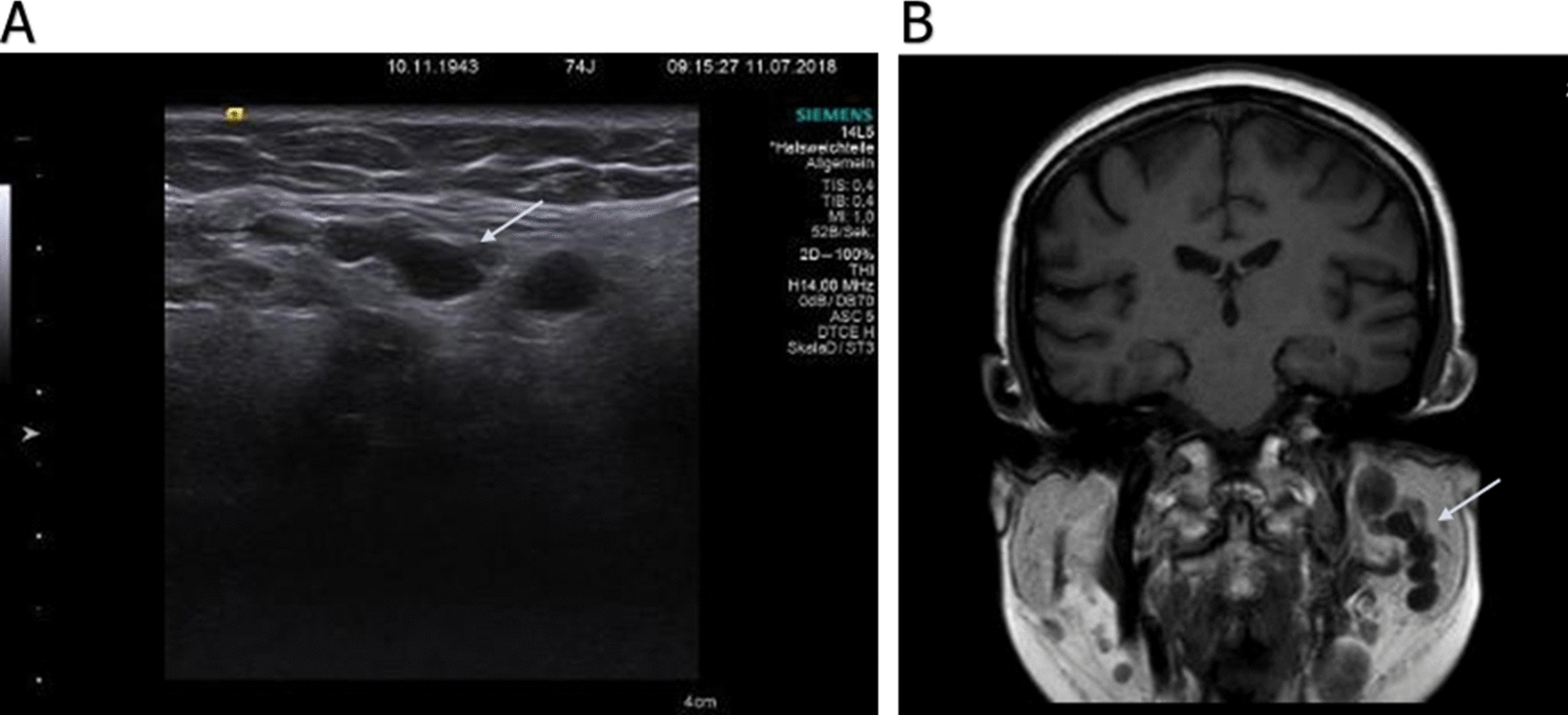
**A** Ultrasonography. Multiple round-shaped, homogeneous and smooth structures along the course of the left marginal mandibular branch of the facial nerve. **B** Cranial magnetic resonance imaging T1 coronal view. Multiple tumors in the left parotid gland along the facial nerve. **A** & **B**: Arrow is showing the tumors

**Fig. 2:**
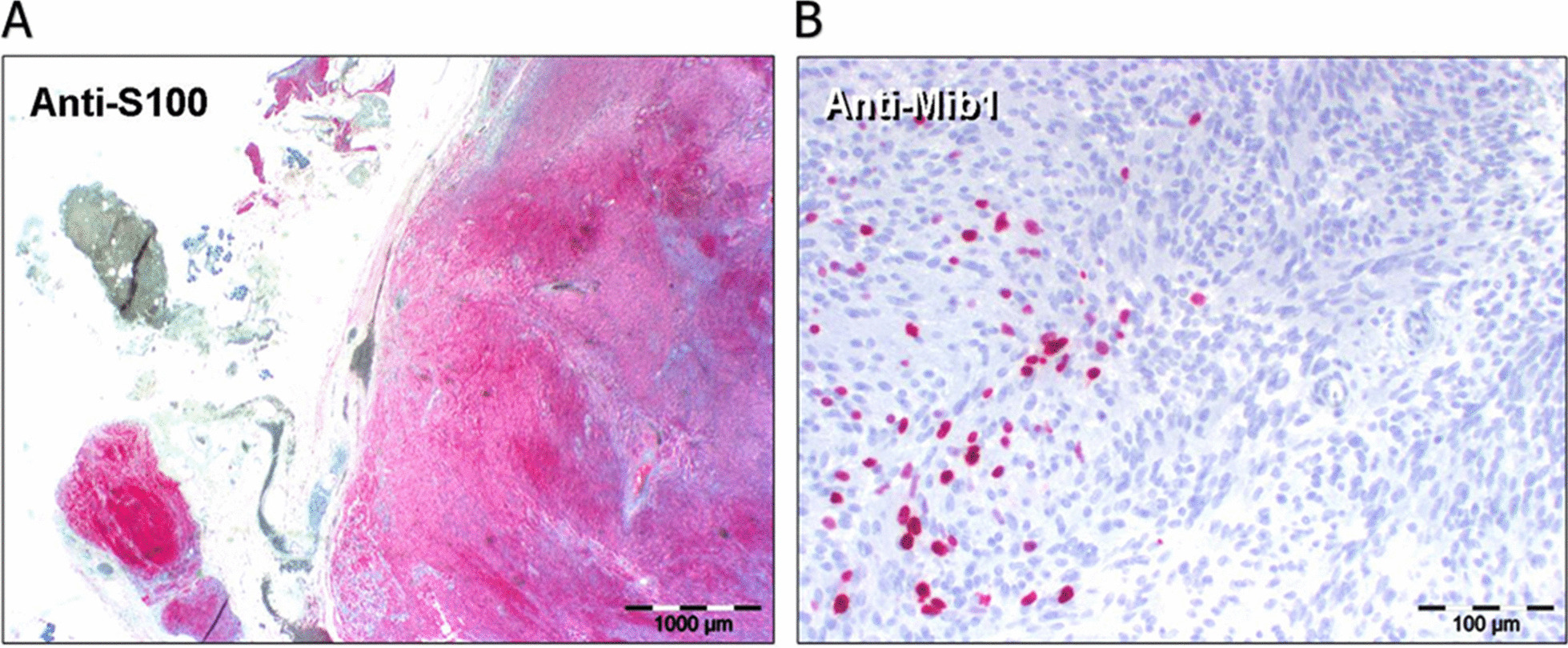
**A** Anti-S100 immunohistochemical staining with strong intralesional signals in nearly all cells. **B** Anti-Mib1 immunohistochemical staining showing several “hot spots” in the tumor tissue

On clinical examination, the facial nerve showed an impaired function of the marginal mandibular branch on the left side as described above with no further functional deficits of the other branches. Eye closure and movement of the forehead muscles were not affected. Ear, nose, and throat routine examination showed no pathological findings. A neurological check-up with a detailed examination of all cranial nerves and sensory and motor function was inconspicuous. All vital signs, including pulse, blood pressure, and temperature, were in physiological range. Initially, cerebral computed tomography (CT) imaging was performed to exclude a cerebral event. It revealed multiple tumors within the left parotid gland. The laboratory blood analysis including inflammation markers [C-reactive protein (CRP), procalcitonin], electrolytes, white and red blood cell counts, liver and renal function markers (AST, ALT, GGT, creatinine, urea), and borreliosis serology showed no conspicuous findings. A high-resolution ultrasound (Fig. 1A) and magnetic resonance imaging (MRI; Fig. 1B) examination of the head without contrast agent were carried out and delineated multiple lesions along the left mandible. They presented as round in shape, well-defined, hypoechoic tumors without a hilar structure, which is reminiscent of lymphoma, but they did not show any hypervascularization or intranodal reticulation. For histological verification, a left-side partial parotidectomy with use of a nerve-integrity monitor and extirpation of an intraparotideal node was performed 7 days after initial presentation at our clinic. We decided to remove the node located at greatest distance from the main trunk of the facial nerve in order to lower the risk of nerve injury. Histomorphological analysis of the resected tissue revealed the diagnosis of a benign schwannoma (Fig. 2). The tumor tissue showed high expression levels of Mib-1 indicating a high proliferation rate as well as S-100 pointing towards a neuroectodermal origin of the tumor cells. After the operation, the patient remained in hospital for five more days before leaving for home without any postoperative complications and without any change of her medication. The size of the nonresected tumors is monitored regularly every 4 months by ultrasonography. The facial nerve function remained unchanged since the operation with a total follow-up of 36 months, and fibromatosis has been excluded by a dermatological work-up including whole-body skin examination and sequencing of the *NF1* and *NF2* gene in the removed tissue.

## Discussion and conclusion

In this work, we describe the clinical case of a patient with multiple schwannomas of the left-sided marginal mandibular branch of the facial nerve, which is the first report of this entity in literature. So far, only a few groups reported on singular schwannomas of the extratemporal segment of the facial nerve mostly associated with neurofibromatosis [1–5]. After diagnostic work-up and an initial working diagnosis of cervical lymphoma, incisional biopsy revealed the diagnosis of multiple Schwannomas in our patient. Lesions are controlled regularly by ultrasound for more than 3 years now and show no signs of progressive growth.

Schwannomas are benign nerve sheath tumors composed of Schwann cells of myelinated peripheral or cranial nerves that are frequently located in the head and neck region but rarely affect the facial nerve [1, 2]. Multiple schwannomas of the marginal mandibular branch of the facial nerve have not been described so far [1, 2]. On ultrasound imaging, schwannomas usually present as singular homogeneous, hypoechoic, round masses, rarely with a pseudocystic appearance or an internal vascular flow [6]. In the present case, multiple tumors were detected, which imitated cervical lymphoma at first glance. Lymphoma is a common malignant disease, and cervical or intraglandular lymph node involvement is frequently seen. The involved lymph nodes are usually located in the submandibular region, upper cervical chain, and posterior triangle regions [7]. Ultrasound is usually able to corroborate the diagnosis. On high-resolution ultrasound, lymphomas show as round in shape, well-defined, hypoechoic tumors with infrequent hilum and/or intranodal reticulation [8]. If there is a clinical and sonographic suspicion of lymphoma, a lymph node extirpation is usually performed to verify the diagnosis. A different surgical procedure is indicated for the suspected diagnosis of schwannoma. As intraparotid facial nerve schwannomas commonly present as slow-growing parotid mass with normal facial nerve function, cervical lymphoma or multiple adenomas of the parotid/submandibular gland could not be ruled out as differential diagnosis based on clinical and radiological examinations; therefore, a surgical biopsy was planned. In this case, we decided in favor of a representative sampling for histological examination instead of a complete removal of all nodes since this would have implied a markedly higher risk for nerve injury. Since diagnostic and therapeutic options markedly differs between parotid gland adenoma, lymphoma, and peripheral nerve schwannoma, all entities need to be considered as differential diagnoses when a patient presents with the aforementioned clinical and radiographic findings, which emphasizes the importance of a careful ultrasonography examination as initial diagnostic work-up. In our case, the patient presented with a palsy of the left lower branches of the facial nerve, which is uncommon for solitary schwannomas and impeded differential diagnosis. One potential explanation for this untypical clinical presentation is the presence of multiple tumors, which in conjunction could have compromised nerve function.

With regard to treatment, options for therapeutic management of multiple schwannomas include surgical therapy [1], radiation [2], off-label immunotherapy [2], or a watch-and-wait strategy [1] with regular clinical examinations and imaging controls depending on the localization and the patient’s individual preferences and course and function of the nerve. To our knowledge, we present so far the first case report of multiple schwannomas of the marginal mandibular branch of the facial nerve. If multiple tumors occur in the parotid gland and the angle of the jaw, this rare differential diagnosis needs to be considered. To plan the right diagnostic surgical intervention and prevent nerve damage, a thorough ultrasound examination is essential in preoperative diagnostic work-up for any suspicious lesion of the parotid gland and jaw region.

## Data Availability

Not applicable.

## Abbreviations

ALT: Alanine transaminase
AST: Aspartate transaminase
CT: Computed tomography
GGT: Gamma glutamyltransferase
MRI: Magnetic resonance imaging
